# Vectors for Genetically-Encoded Tags for Electron Microscopy Contrast in *Drosophila*

**DOI:** 10.1186/s12575-016-0034-1

**Published:** 2016-02-01

**Authors:** Marco Man Kin Tsui, Anri Itoh, Mohamed Amgad, Shao-Fang Wang, Toshio Sasaki

**Affiliations:** Developmental Signalling Unit, Research Lab 1, Level C, Okinawa Institute of Science and Technology, Tancha 1919-1, Onna-son, Kunigami-gun, 904-0412 Okinawa Japan; Faculty of Medicine, Cairo University, Cairo, Egypt

**Keywords:** Electron microscopy, Imaging, Drosophila, Mini-SOG

## Abstract

**Background:**

One of the most notable recent advances in electron microscopy (EM) was the development of genetically-encoded EM tags, including the fluorescent flavoprotein Mini-SOG (Mini-Singlet Oxygen Generator). Mini-SOG generates good EM contrast, thus providing a viable alternative to technically-demanding methods such as immuno-electron microcopy (immuno-EM). Based on the Mini-SOG technology, in this paper, we describe the construction, validation and optimization of a series of vectors which allow expression of Mini-SOG in the *Drosophila melanogaster* genetic model system.

**Findings:**

We constructed a Mini-SOG tag that has been codon-optimized for expression in *Drosophila* (DMS tag) using PCR-mediated gene assembly. The photo-oxidation reaction triggered by DMS was then tested using these vectors in *Drosophila* cell lines. DMS tag did not affect the subcellular localization of the proteins we tested. More importantly, we demonstrated the utility of the DMS tag for EM in *Drosophila* by showing that it can produce robust photo-oxidation reactions in the presence of blue light and the substrate DAB; the resultant electron micrographs contain electron-dense regions corresponding to the protein of interest. The vectors we generated allow protein tagging at both termini, for constitutive and inducible protein expression, as well as the generation of transgenic lines by P-element transformation.

**Conclusions:**

We demonstrated the feasibility of Mini-SOG tagging in *Drosophila*. The constructed vectors will no doubt be a useful molecular tool for genetic tagging to facilitate high-resolution localization of proteins in *Drosophila* by electron microscopy.

**Electronic supplementary material:**

The online version of this article (doi:10.1186/s12575-016-0034-1) contains supplementary material, which is available to authorized users.

## Introduction

**Fig. 1:**
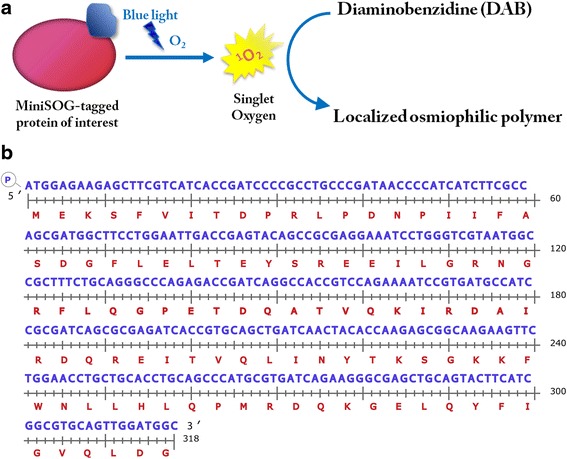
Principle behind- and gene synthesis of- the codon-optimized *Drosophila*-specific Mini-SOG (DMS). **a** Schematic diagram demonstrating the principle behind EM contrast generation using Mini-SOG. **b**. DNA sequence and the associated translation of DMS

The recent development of genetically-encoded tags for electron microscopy such as Mini-SOG [1] and APEX [2] provides a way to simplify detection and localization of proteins using electron microscopy (EM). Mini-SOG (Mini-Singlet Oxygen Generator), developed by the Tsien group, is a fluorescent flavoprotein that was originally developed by genetic engineering of *Arabidopsis* phototropin 2. Mini-SOG creates EM contrast by generating oxygen singlets when activated by light, which in turn catalyze the conversion of diaminobenzidine (DAB) to a localized osmiophilic polymer [1] (Fig. 1a). The electron microscopic contrast generated by DAB polymer has low diffusibility due to extensive cross-linking, and the staining is spatially adjacent to the protein of interest, and not separated by several nanometers as is the case with immunolabelling [1, 3]. This method has been successfully employed in human and *C. elegans* cells [1], yet there have been no accounts so far on its use in other model organisms. In this study we constructed a set of vectors for Mini-SOG tagging in *Drosophila*, and validated and optimized its use in this model organism.

## Materials and Methods

### Plasmid Construction

Open reading frame of *Drosophila* Histone2Av (CG5499) was amplified from cDNA prepared from *Drosophila* embryos. Mitochondrial-targeting sequence corresponding to the N-terminal 31-amino acid mitochondrial import signal of cytochrome c oxidase subunit VIII (CG7181) [4] was amplified from adult *Drosophila* genome. The DNA sequence of Mini-SOG (based on Shu et al., 2011 [1]), codon-optimized for Drosophila expression, and the associated primer sequences (fourteen 40-mers, named DMS1–DMS14) used for gene assembly, were designed using the online program DNAWorks v3.2.2 (https://hpcwebapps.cit.nih.gov/dnaworks/, National Insitute of Health, USA), with codon frequency threshold set to 20 %. The DMS gene was synthesized by a two-step PCR method [5, 6] . The resultant fragment was subcloned into pUbi as pUbi-C-DMS. All other DMS vectors were also constructed by restriction enzyme cloning. The sequences of the primers used are detailed in Additional file 1: Table S1 and the maps of the vectors are in Additional file 1: Figure S2. The sequences of all constructs were confirmed by Sanger sequencing.

### Cell Culture and Transfection

*Drosophila* cell lines were maintained according to standard protocols. Cells were transfected with 0.1–0.3 ug DNA using Effectene Transfection Reagent (QIAGEN) according to the manufacturer’s protocol. For induction of the metallothionein promoter, 0.2–0.5 mM copper sulphate (final concentration) was added 24 h post-transfection. Two days following transfection, the cells were harvested for confocal microscopy and photo-oxidation.

### Mitotracker Staining

Cells were incubated with 250 nM of Mitotracker Red (Molecular Probes) in culture medium for 30 min at room temperature, washed once with medium and immediately observed under a microscope.

### Confocal Microscopy

Transfected cells were plated onto gridded 35 mm glass-bottom dishes (P35G-0-14-C, Matek Corp., USA) for 30 min, and observed under the microscope, either directly or after fixation (as described below). Confocal images were taken using LSM 780 (ZEISS, Germany).

### Photo-Oxidation

Photo-oxidation by Mini-SOG was performed essentially as described by Shu et al., 2011 [1] with a few modifications. Cells were fixed by 2.5 % glutaldehyde in 0.1 M cacodylate buffer for an initial 5 min at room temperature, followed by a one-hour incubation period on ice. Cells were then washed five times with 0.1 M cacodylate buffer and incubated with blocking buffer (50 mM glycine, 10 mM KCN and 5 mM aminotriazole in 0.1 M cacodylate buffer) for 30 min. Afterwards, the buffer was replaced with oxygen-saturated DAB (0.1 mg/ml DAB in 0.1 M cacodylate buffer). Samples were then illuminated with a 63× oil objective using a high pressure mercury lamp with the CFP filter set (EX436/20. DM455. BA480/40) for 2–6 min, depending on the expression levels of the proteins. After photo-oxidation, cells were washed five times with 0.1 M cacodylate buffer and sent immediately for electron microscope sample preparation.

### Electron Microscopy

Samples were prefixed with 2.5 % glutraldehyde in 0.1 M sodium cacodylate buffer, postfixed with 1 % osmium tetroxide (OsO_4_) in 0.1 M sodium cacodylate buffer for 30 min, and then washed with pure water three times. The samples were subsequently dehydrated by ethanol series. A 1:1 ethanol:epoxy resin mix was then added to the samples and incubated on a mixer for 30 min. Afterwards, the resin mix was replaced by pure resin and incubated overnight, and an extra change of pure resin was performed. The resin was then allowed to polymerize in a 60 ° C oven for two days. The resin block was trimmed to 0.5 mm cubes, which were further sliced to 50 nm sections using a microtome. These sections were mounted onto copper grids (MAXTAFORM HF34, 200 holes) without any stain and observed by a JEM-1230R microscope (JEOL), at an accelerating voltage of 100 keV, using bright field imaging.

## Results and Discussion

**Fig. 2:**
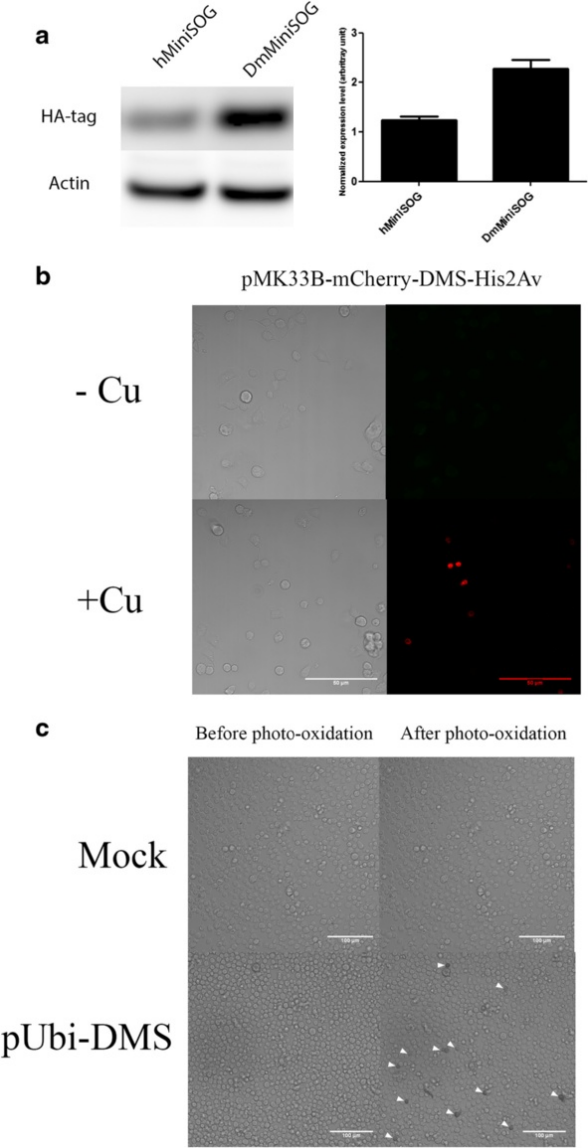
Enhanced expression of codon optimized MiniSOG in Drosophila which generates robust EM contrast after photo-oxidation reaction. **a**. Western blot of cell extracts prepared from cells transfected with equal amounts of original human MiniSOG (hMiniSOG, left lane)[1] and Drosophila codon optimized MiniSOG (DmMiniSOG or DMS, right lane) probed with anti-HA antibodies (*upper panel*) and anti-actin antibodies (*lower panel*) as a loading control. **b**. Bar chart showing the normalized expression levels (HA/actin ratio) for hMiniSOG and DmMiniSOG from three independent experiments. DmMiniSOG shows an 83 % increase in expression level compared to hMiniSOG. **c**. inducible expression of DMS fusion proteins by metallothionein promoter. Cells transfected with pMK33B-mCherry DMS-His2Av construct were observed under confocal microscopy. Robust expression of the DMS-His2Av fusion was only observed after 0.5 M copper sulphate was added to the culture (lower right panel, mCherry chanel) whereas no expression in cells without cupper induction (upper right panel). **d**. High-contrast dark precipitate observed in cells transfected with pUbi-DMS after photo-oxidation (lower right panel, dark arrows) but not in mock transfected cells (upper panels), under a light microscope

**Fig. 3:**
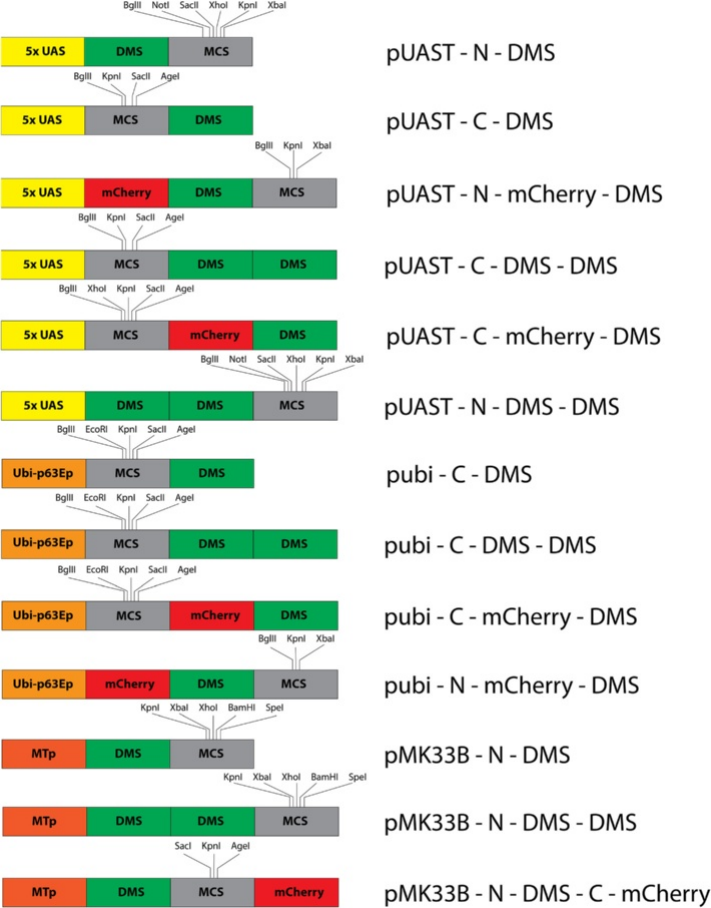
Features of DMS vectors generated in this study. Abbreviations: *UAS*, upstream activating sequence (from pUAST); *MTp*, metallothionein promoter (derived from pMK33B); *pubi*, Ubi-p63E promoter; *DMS*, Drosophila Mini-SOG; *MCS*, multiple cloning sites. Unique restriction enzyme sites for cloning are indicated above the MCS box in each vector

First, we designed sequences that optimize the translation efficiency in *Drosophila* cells (Fig. 1b). A triple hemagluttin (HA3) tag was added to the N-terminus of the original and the optimized sequences separately. Equal amounts of each plasmid were transfected into Kc167 cells (described below) and the expression level of each construct was measured by western blot using anti-HA antibodies (Fig. 2a, left panel). There is a considerable enhancement of expression of the codon-optimized MiniSOG (DmMiniSOG) compared to the original MiniSOG construct (hMiniSOG) in *Drosophila* cells. After normalization using actin levels of the cell extracts, DmMiniSOG showed a 100 % increase in protein level compared to hMiniSOG (Fig. 2a, right panel). From this point onward, we will refer to DmMiniSOG as DMS for short. The assembled DMS gene was then subcloned behind an ubiquitin promoter as pUbi-C-DMS (Fig. 3). *Drosophila* Clone 8 and Kc167 cells were then transfected with the resultant construct and photo-oxidation was carried out under blue-light illumination in the presence of DAB. Clone 8 line is derived from third instar larval imaginal discs cells, whereas Kc167 lines are embryonic cells from 8 to 10 h old embryos and have plasmatocyte properties [7]. These cell lines were chosen because they have been extensively exploited in *Drosophila* research, and have well-established protocols for culturing, transfection and immunostaining. A dark, highly contrasting, osmiophilic precipitate was observed in cells transfected with DMS construct, but not in mock transfected cells (Fig. 2b), demonstrating the photo-oxidative capability of DMS in *Drosophila* cells. We subsequently generated a series of DMS expression vectors to incorporate different modularities. Fig. 3 shows the essential features of this set of vectors.

We engineered the DMS tag at both the N- terminal and C- terminal ends. In addition to the monomeric DMS tag, a tandem DMS dimer was constructed to further enhance the enzymatic efficiency of the tag. There are three choices of promoters: constitutive (ubquitin promoter) and inducible (metallothionein and UAS promoters). The ubiquitin promoter is derived from the Ubqi-p63E gene (CG11624), which drives high level expression in cell lines and tissues in *Drosophila* flies [8]. We also included a mCherry tag to the DMS vector. Hygromycin gene in pMK33b backbone allows for stable clone generation in cell line while pUAST backbone can be used for the generation of transgenic flies [9]. In addition, the metallothionein promoter in pMK33b allows inducible expression in cell culture. As shown in Fig. 2b, without the addition of copper sulphate, cells transfected with the pMK33B-mCherry-DMS-His2Av constructs showed no red fluorescence and hence no expression of the mCherry-DMS-His2Av fusion protein (Fig. 2c, upper panel). After the addition of 0.5 mM copper sulphate for 48 h, robust expression of mCherry-DMS-His2Av was detected by red nuclear fluorescence (Fig. 2c, lower panel). Altogether, we constructed 14 DMS vectors that should prove to be suitable for a wide variety of needs in EM studies using the *Drosophila* model system.

**Fig. 4:**
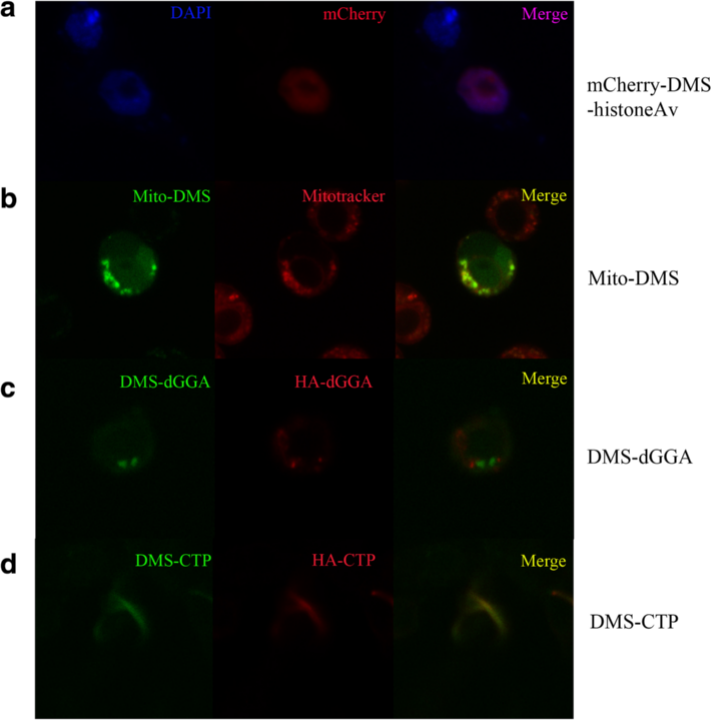
Various DMS-fusion proteins show proper localization. **a**-**d** Confocal microscopy of *Drosophila* Clone 8 cells transfected with various DMS fusion constructs: **a** mCherry-DMS-Histone2Av. From left to right, DAPI channel, mCherry channel and merged image. **b**. Mito-DMS. From left to right, FITC channel, mCherry channel (mitotracker) and merged image. **c**. DMS-dGGA. From left to right, FITC channel, mCherry channel (anti-HA staining) and merged image. **d**. DMS-CTP. From left to right, FITC channel, mCherry channel (anti-HA staining) and merged image

Next we set out to test whether the DMS tags affect protein localization in Clone-8 cells. We generated DMS fusion to histone 2Av (a nuclear marker [10]), a chimera protein consisting of the mitochondrial import signal from Co VIII [11] attached to the N-terminus of DMS (pMK33B-N-DMS) and DMS fusion proteins to the Drosophila Golgi-localized, gamma-adaptin ear containing binding protein (dGGA [12]) and to the enzyme CTP synthase [13, 14]. As can be seen in Fig. 4a, the mCherry-DMS tagged histone2Av resides in the nucleus. Mito-DMS had punctate distributions in cells as previously reported [1, 15] and was found to co-localize with mitochondrial marker Mitotracker Deep Red [16] (Fig. 4b). From Fig. 4c, it can be seen that DMS-dGGA exists as punctate structures which are in juxtaposition with the cis-Golgi marker dGM130 (red fluorescence), which is consistent with the localization properties of endogenous dGGA ([12]). DMS-CTP synthase forms long filamentous cellular structures (called cytoophia) similar to the endogenous protein and shows complete overlap with HA-tagged CTP synthase (Fig. 4d). We therefore concluded that the DMS tag does not affect the localization of the proteins tested.

**Fig. 5:**
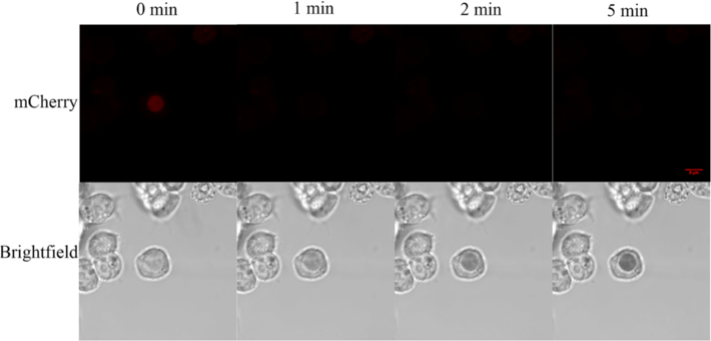
DMS promotes robust photo-oxidation reaction in *Drosophila* cells. *Upper panel:* Confocal images of Clone 8 cells transfected with mCherry-DMS-Histone2Av, before photo-oxidation (leftmost) and after photo-oxidation (second to fourth figures from the left), with the duration of photo-oxidation (in minutes) indicated above the panel. *Lower panel:* Corresponding bright field images. Note the disappearance of mCherry signals and the accompanying appearance of the dark deposit in the nucleus of the transfected cells

We subsequently investigated the time dependence of the photo-oxidation reaction by DMS in *Drosophila* cells, using histone2Av fusion. We found that the bleaching of mCherry occurs after around 1 min of reaction, while the photo-oxidation reaction continues to be very robust 4 min after the reaction started, as judged by the continuous increase in the darkness of the osmium deposit in the nucleus (Fig. 5, lower panel). This indicates that under correct fixation and photo-oxidation reaction conditions, the DMS fusion can sustain a long period of reaction. This sustained reaction should prove to be particularly useful when the DMS-fusion construct shows lower expression levels.

**Fig. 6:**
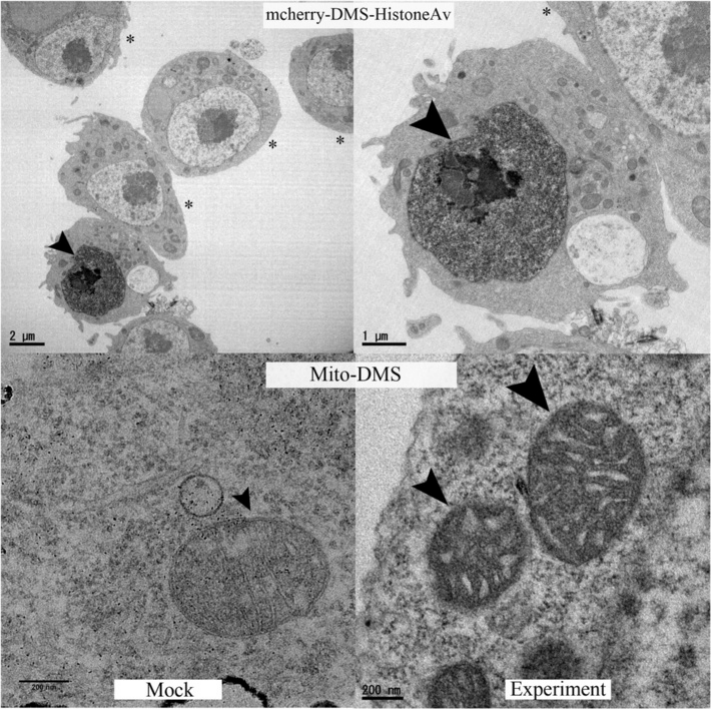
DMS produces strong EM contrast in *Drosophila* cells. *Upper panel:* Electron micrographs of Clone 8 cells transfected with mCherry-DMS-Histone2Av. The arrowhead points to the stained nucleus of a transfected cell, while untransfected cells are marked with asterisks. The image on the right is a higher magnification version of the one on the left. *Lower panels:* A cell transfected with mito-DSM. Arrowheads point to mitochondria. The left image shows a cell with mock photo-oxidation reaction with DAB omitted, and the right image shows a cell with heavily stained mitochondria resulting from robust photo-oxidation reaction

Next we looked at mCherry-DMS-histone2Av and mito-DMS cells under the electron microscope. It can be clearly seen (Fig. 6, upper panel) that the cells transfected with histone2Av show high EM contrast in the nucleus (marked by arrowheads), particularly in the chromatin where the majority of histone2A resides. On the other hand, untransfected cells (marked by asterisks) show very low EM contrast in the nucleus. Furthermore, we observed heavy EM staining in the lumen of the mitochondria of cells harboring mito-DMS (Fig. 6, lower right panel) compared to those cells treated with mock reaction (Fig. 6, lower left panel).

**Fig. 7:**
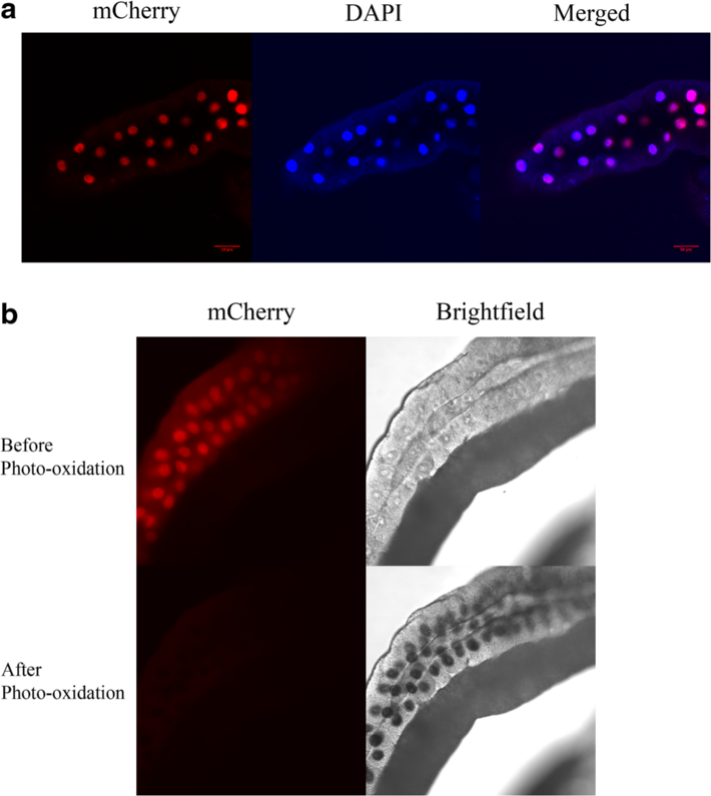
DMS promotes robust photo-oxidation reaction in *Drosophila* tissue. **a** Confocal images of L3 salivary glands expressing mCherry-DMS-Histone2Av. Left: mCherry, middle: DAPI and right: merged. **b** *Upper panel:* Images of L3 salivary glands expressing mCherry-DMS-Histone2Av before photo-oxidation. Left: mCherry channel, right: brightfield image. *Lower panel:* Corresponding images after a 10 min photo-oxidation reaction

Lastly we checked if the DMS tag works in *Drosophila* tissues. A transgenic line was made by transgenesis using the pUAST-mCherry-DMS-his2Av construct. This line expresses mCherry-DMS-his2Av in the salivary gland and wing discs when crossed to the MS1096 GAL4 driver. Salivary glands from L3 larvae expressing mCherry-DMS-his2Av were dissected, fixed and stained with DAPI. As can be seem from Fig. 7a, mCherry-DMS-his2Av completely co-localize with DAPI staining. We then performed photo-oxidation in the salivary gland. After 10 min of photo-oxidation reaction in the salivary gland, robust dark precipitate was observed in the nuclei (Fig. 7b, lower right panel, compared to before photo-oxidation, Fig. 7b, upper right panel). Hence photo-oxidation in *Drosophila* tissue is by DMS tag is robust.

A number of factors should be kept in mind while using of vectors described in this report:

- Expression level:
  - Over-expression sometimes leads to the mis-localization of the tagged protein. Reducing expression levels may be done in several ways. For the MT promoter, reducing the amount of copper added by titration may be enough to reduce expression levels for correct localization. Another way to get around this is to switch existing promoters with a weaker substitute.
- Reaction time:
  - Enough time should be left for a suitable amount of oxidation product to form However, bear in mind that overly extended reactions lead to excessive buildup of free radicals, damaging the internal cellular structure.
- Terminal fusion site:
  - As with any other exogenous protein tagging method, there is a chance that the DMS tags will affect trafficking and localization of the proteins being investigated. Hence, it is advisable to make both N-terminal and C-terminal tagged fusions, then to choose constructs yielding the most natural expression and trafficking. We created vectors for fusion at both ends for this purpose. Typically, the localization of the endogenous protein could be investigated in a separate immunolabelling control experiment under light microscopy to check for proper localization of the fusion protein before starting the EM experiment.

In conclusion, we constructed a set of vectors for optimized Mini-SOG mediated photo-oxidation in *Drosophila* cells for high resolution EM detection of proteins. These vectors are inducible and can be used for cell culture as well as transgenic preparations. We believe that this vector set will prove to be a useful resource not only to the *Drosophila* community, but also to anyone interested in using *Drosophila* as a disease model system in general.

## Additional File

Additional file 1:
**Supplementary Table 1. Primer sequences.** Supplementary Figure 2. Plasmid Maps (DOCX 1425 kb)
