# Supplementary material for: Vectors for Genetically-Encoded Tags for Electron Microscopy Contrast in Drosophila
Source: Biol Proced Online. 2016 Feb 1;18:5. doi: 10.1186/s12575-016-0034-1 (PMC4736618; doi:10.1186/s12575-016-0034-1)

**Supplementary Table 1. Primer sequences**

| **Primer name** | **Sequence (5’-3’)** |
| --- | --- |
| Dm-miniSOG-Age1-F | ACCAACCGGTCGCCACCATGGAGAAGAGCTTCGTCAT |
| Dm-miniSOG-STOP-Xba1-R | ACCATCTAGATTAGCCATCCAACTGCACGCCGA |
| DMS-C-Nco1-R | ACCACCATGGACCCGCCACCACTCCCGCCACCAGAGCCATCCAACTGCACGCCGA |
| DSM-C-BglII-R | ACCAAGATCTGAGTCCGGAGCCATCCAACTGCACGCCGA |
| DSM-C-Nco1-F | ACCACCATGGAGAAGAGCTTCGTCAT |
| DSM-C-Not1-F | ACCAGCGGCCGCAACATGGAGAAGAGCTTCGTCAT |
| DSM-long-MCS-R | ACCAACTAGTCTCGAGTCTAGAGGTACCAGATCTGAGTCCGGAGCCATCCAACTGCACGCCGA |
| DSM-noNco1-Age1-F | ACCAACCGGTCGCATGGAGAAGAGCTTCGTCAT |
| DSM-noNco1-EcoR1-F | ACCAGAATTCATGGAGAAGAGCTTCGTCAT |
| DSM-Not1-Inframe-F | ACCAGCGGCCGCATGGAGAAGAGCTTCGTCAT |
| DSM1 | ATGGAGAAGAGCTTCGTCATCACCGATCCCCGCCTGCC |
| DSM2 | GCTGGCGAAGATGATGGGGTTATCGGGCAGGCGGGGATCG |
| DSM3 | CCCATCATCTTCGCCAGCGATGGCTTCCTGGAATTGACCG |
| DSM4 | GGATTTCCTCGCGGCGTACTCGGTCAATTCCAGGAAGCC |
| DSM5 | CAGCCGCGAGGAAATCCTGGGTCGTAATGGCCGCTTTCTG |
| DSM6 | CTGATCGGTCTCTGGGCCCTGCAGAAAGCGGCCATTACGA |
| DSM7 | GGCCCAGAGACCGATCAGGCCACCGTCCAGAAAATCCGTG |
| DSM8 | CGCGCTGATCGCGGATGGCATCACGGATTTTCTGGACGGT |
| DSM9 | TCCGCGATCAGCGCGAGATCACCGTGCAGCTGATCAACTA |
| DSM10 | AACTTCTTGCCGCTCTTGGTGTAGTTGATCAGCTGCACGG |
| DSM11 | CCAAGAGCGGCAAGAAGTTCTGGAACCTGCTGCACCTGCA |
| DSM12 | CGCCCTTCTGATCACGCATGGGCTGCAGGTGCAGCAGGTT |
| DSM13 | GCGTGATCAGAAGGGCGAGCTGCAGTACTTCATCGGCGTG |
| DSM14 | GCCATCCAACTGCACGCCGATGAAGTACTGC |
| His2A-Bglll F | ACCAAGATCTATGTCTGGACGTGGAAAAGG |
| His2A-S-Xbal R | ACCATCTAGATTAGGCCTTCTTCTCGGTCT |
| His2av-Nco1-F | ACCACCATGGCTGGCGGTAAAGCAGG |
| His2av-Xba1-R | ACCATCTAGATTAGTAGGCCTGCGACAGAA |
| mCherry-BglII-F | ACCAAGATCTCACCATGGTGAGCAAGGGCGAGGA |
| mCherry-EcoR1-F | ACCAGAATTCATGGTGAGCAAGGGCGAGGA |
| mCherry-EcoR1-F | ACCAGAATTCATGGTGAGCAAGGGCGAGGA |
| mCherry-Not1-R | ACCAGCGGCCGCCCTTGTACAGCTCGTCCATGC |
| mito-BglII-F | ACCAAGATCTATGTTCCAAAACAGCGCTGC |
| mito-Kpn1-R | ACCAGGTACCGCGGTCGGAGGTCCAGAGACGA |
| Ubi-pro-EcoR1 R | ACCAGAATTCTTGGATTATTCTGCGGGA |

**Supplementary Figure 2. Plasmid Maps**


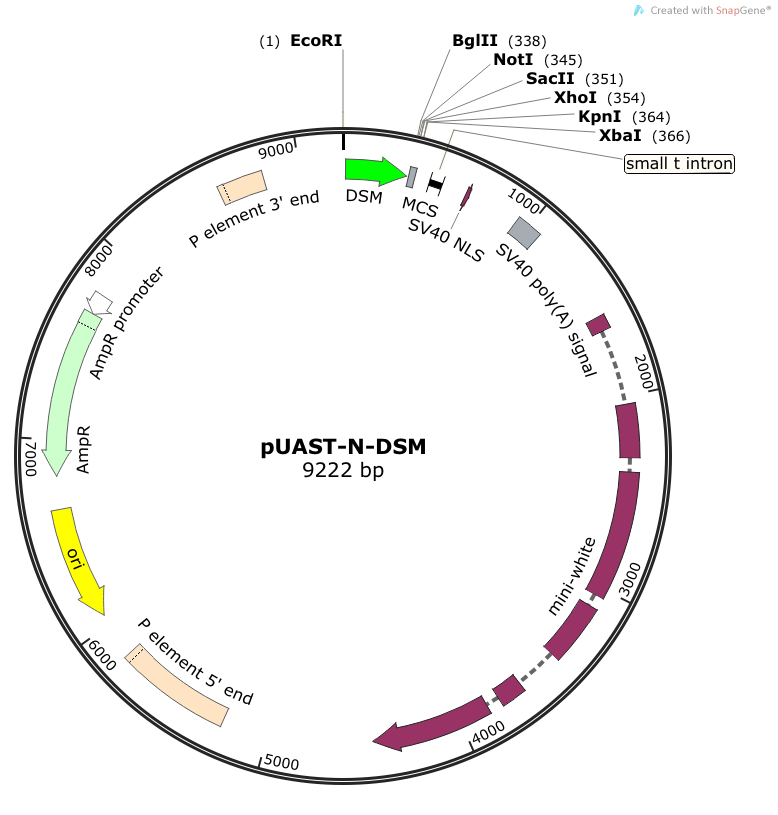


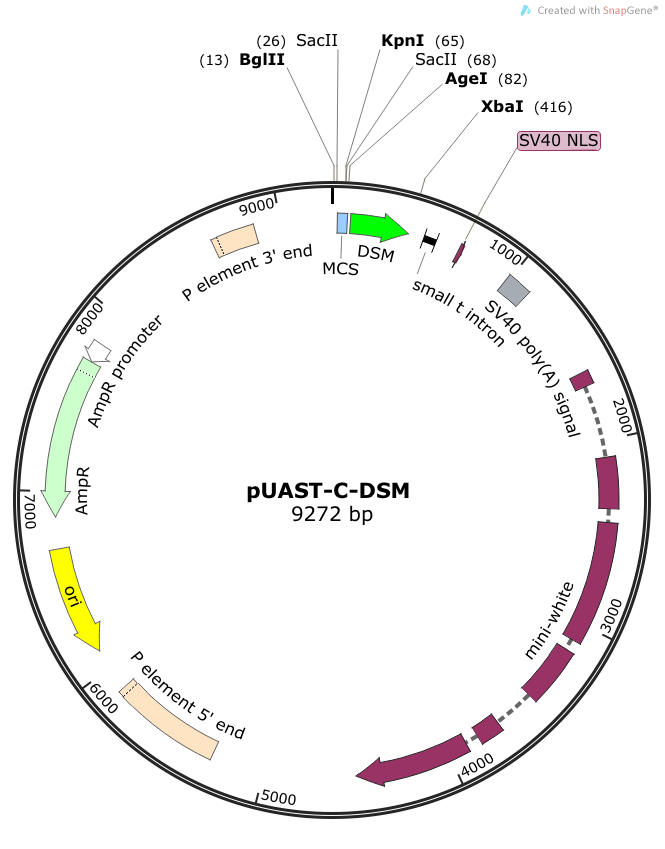


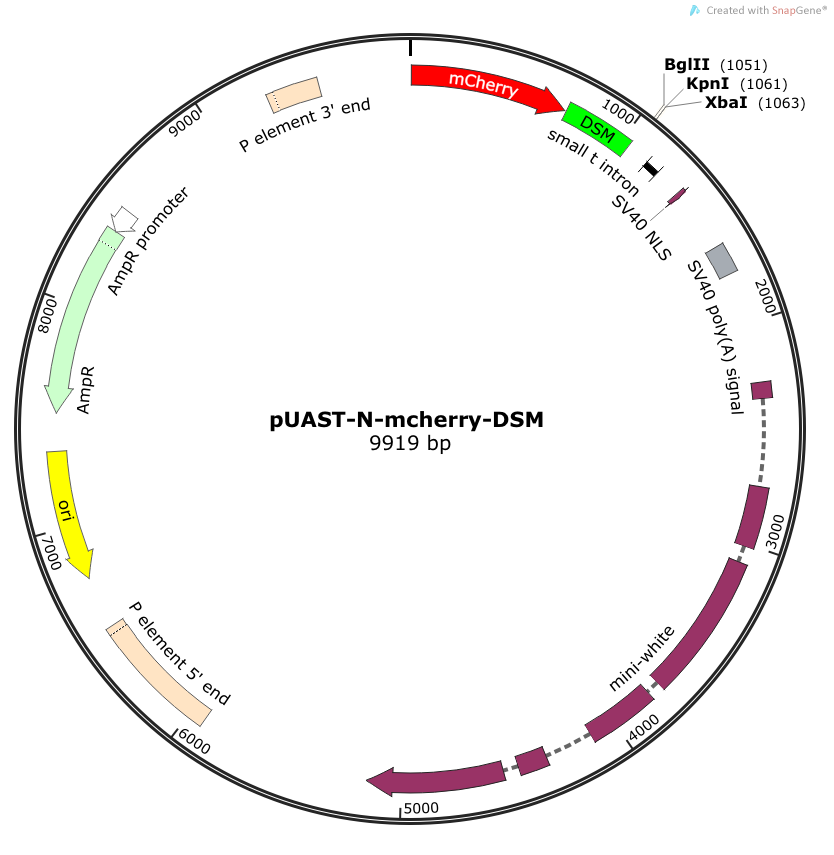


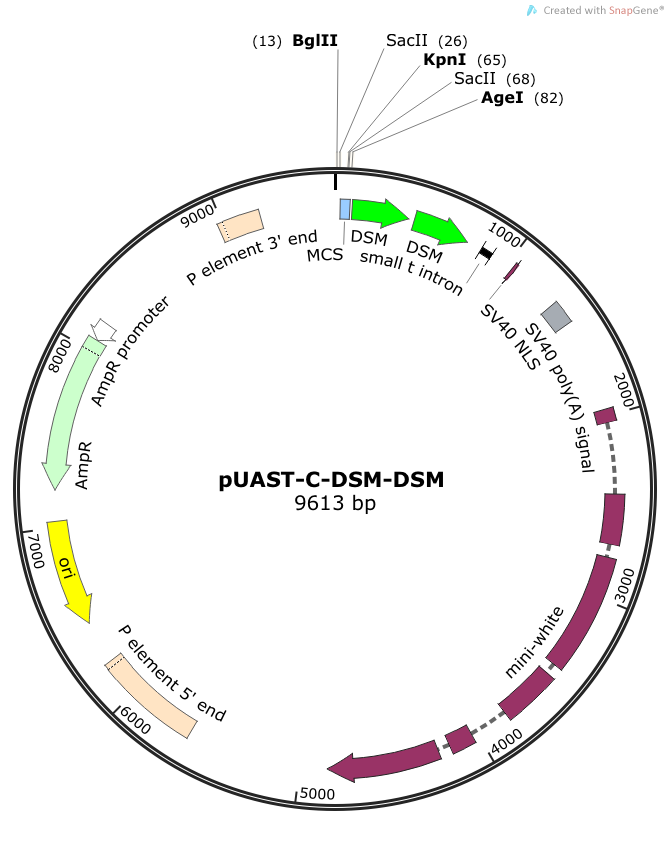


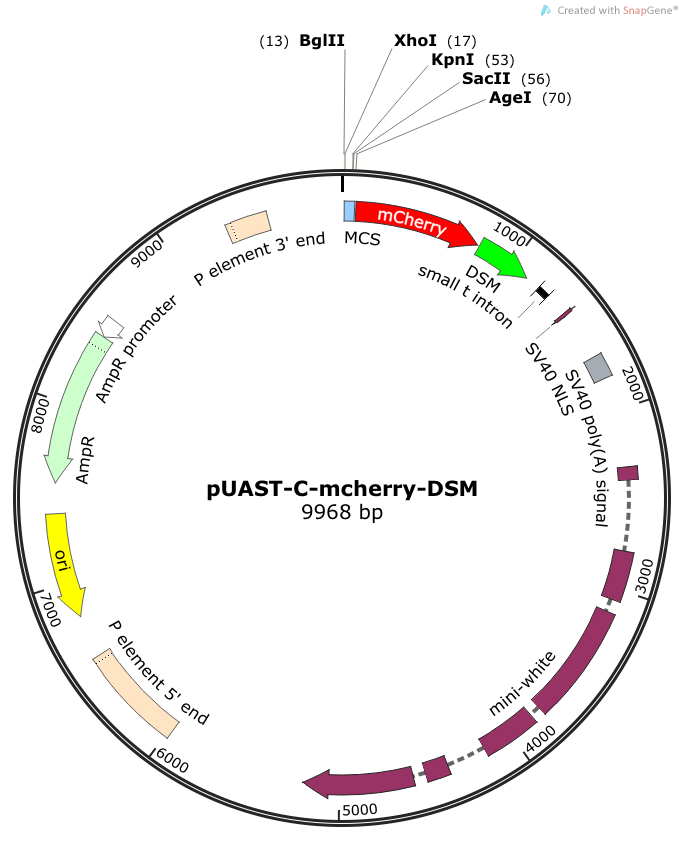


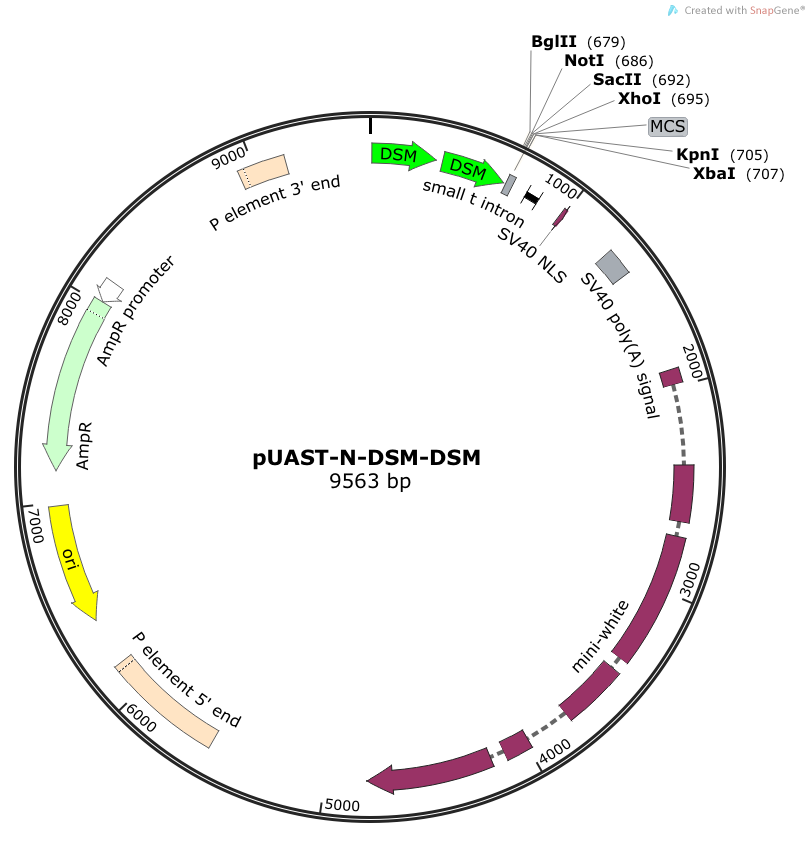


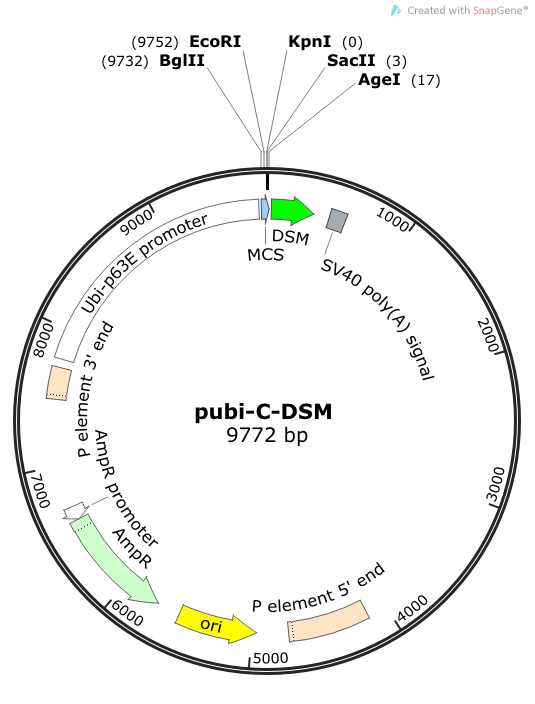


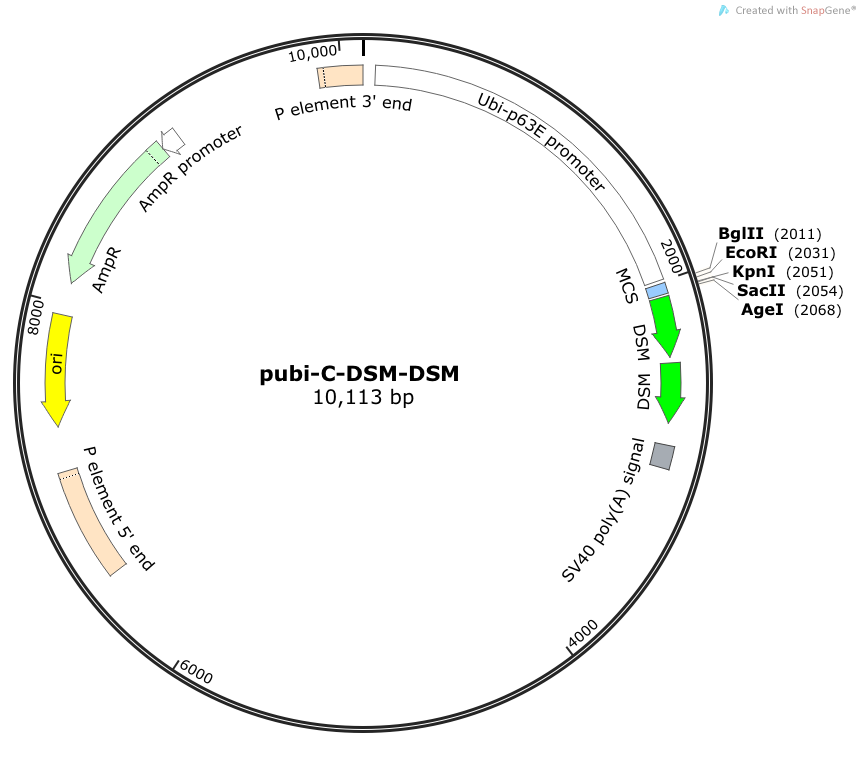


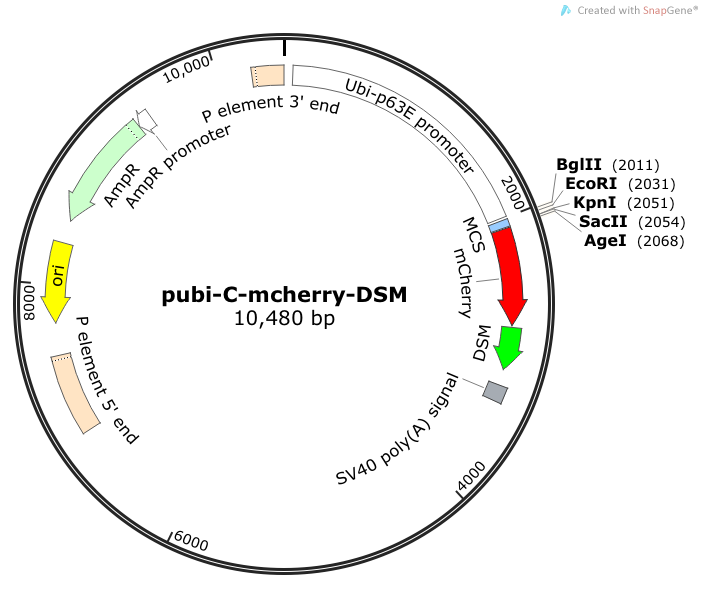


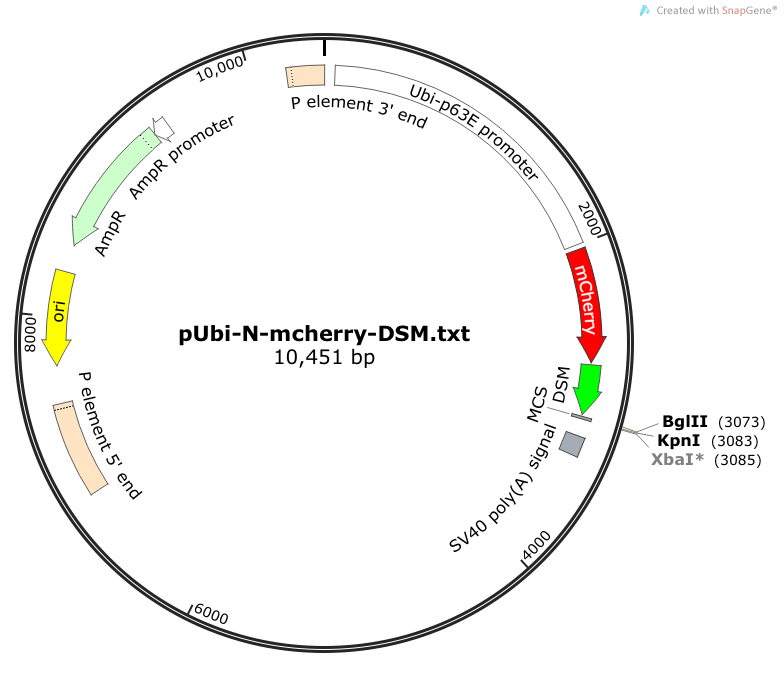


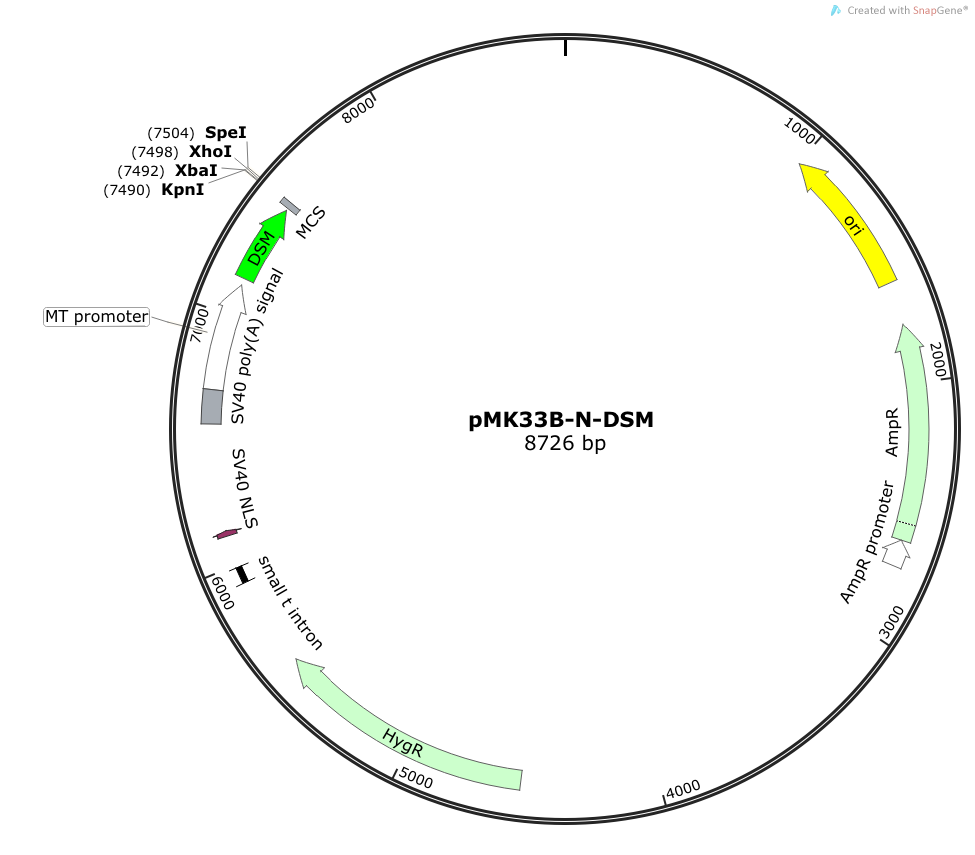


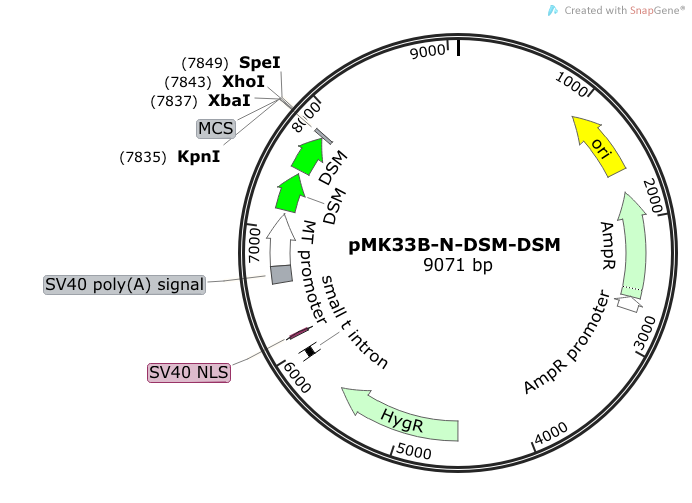


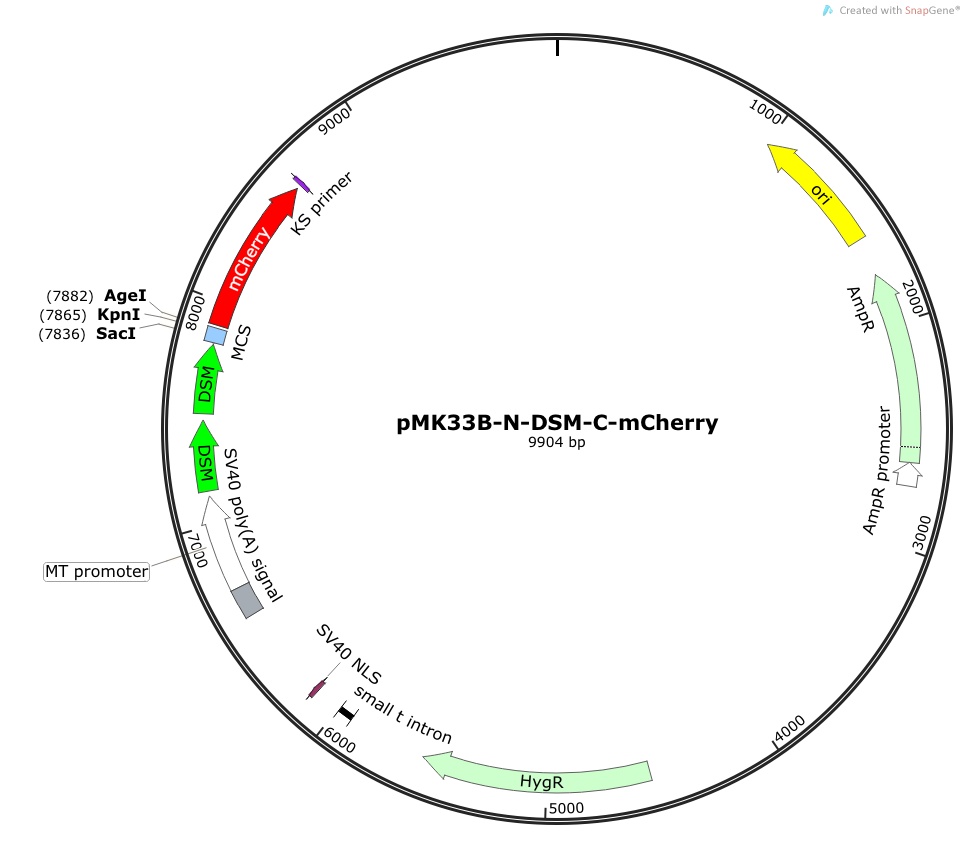

Supplement: Additional file 1: — Supplementary Table 1. Primer sequences. Supplementary Figure 2. Plasmid Maps (DOCX 1425 kb) [file 12575_2016_34_MOESM1_ESM.docx]
